# Microwave alkylation of lithium tetrazolate

**DOI:** 10.1007/s00706-016-1867-7

**Published:** 2016-11-30

**Authors:** Danny Müller, Christian Knoll, Peter Weinberger

**Affiliations:** Institute of Applied Synthetic Chemistry, TU Wien, Getreidemarkt 9/163-AC, 1060 Vienna, Austria

**Keywords:** N1-Tetrazole, Heterocycles, Basicity, Solvent effect, N-Heterocyclic ligands, Spin crossover

## Abstract

**Abstract:**

N1-substituted tetrazoles are interesting ligands in transition metal coordination chemistry, especially in the field of spin crossover. Their synthesis is performed in most cases according to the Franke-synthesis, using a primary amine as reagent introducing the substitution pattern. To enhance flexibility in means of substrate scope, we developed a new protocol based on alkylation of lithium tetrazolate with alkyl bromides. The N1–N2 isomerism of the tetrazole during the alkylation was successfully suppressed by use of highly pure lithium tetrazolate and 30 vol.% aqueous ethanol as solvent, leading to pure N1-substituted products. The feasibility of this reaction was demonstrated by a selection of different substrates.

**Graphical abstract:**

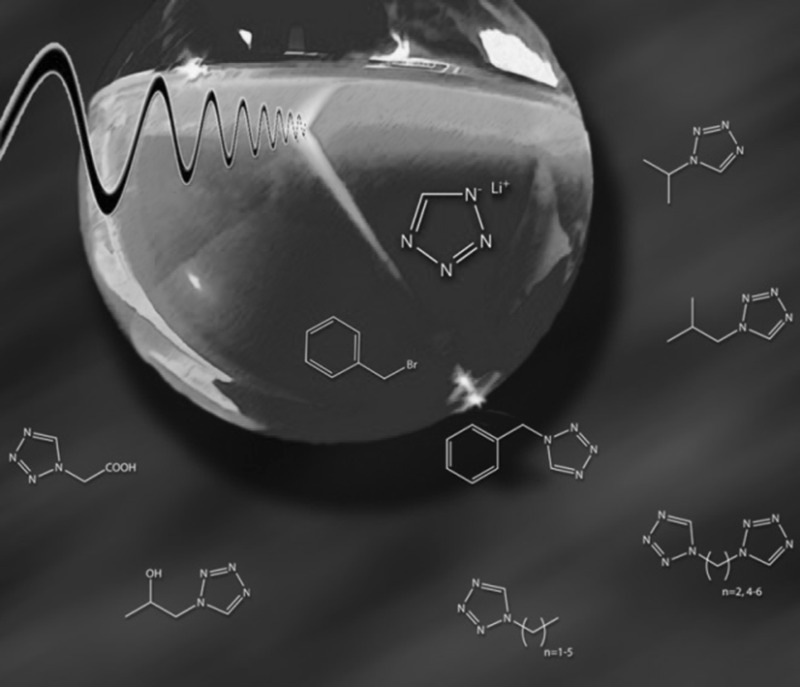

## Introduction

Tetrazoles are five-membered 6π-aromatic heterocyclic compounds and, in the case of the unsubstituted form, with the notably high nitrogen content of 79.98%. Tetrazoles allow for three different isomers (Scheme 1), among which only the 5*H*–tetrazole being non-aromatic [1].

**Figure:**
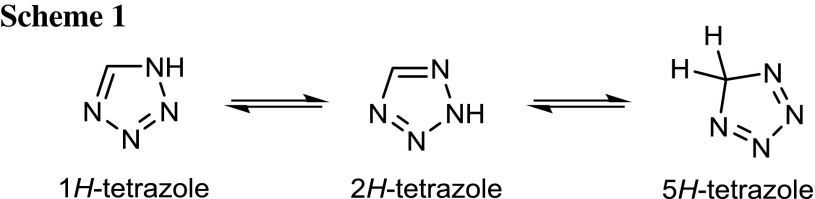

Depending on the substituent, substituted tetrazoles can undergo several forms of tautomerism: apart from the ring-opening within the azido-imine-tautomerism, C5-alkyl or unsubstituted rings undergo changes in the substitution pattern via annular-tautomerism [2], whereas an electronegative substituent in the C5-position favours amino-imino-tautomerism [3].

The very first reported employments of tetrazoles were as colorants, photographical chemicals, explosives, propellants, and agrochemicals [4–6]. Since the 1960s, tetrazoles witnessed a renaissance but now with their application strongly depending on their isomeric form. In the medicinal chemistry, N2- and especially C5-substituted tetrazoles are used as carboxylic acid bioisosteres [7], as structural motif for angiotensin blockers [8–10], as activator in oligonucleotide synthesis [11] or in the MTT-assay, determining the metabolic cell activity by reduction of a tetrazole-dye to a formazan by living cells [12].

In contrast, N1-substituted tetrazoles are mainly used as ligands in inorganic coordination chemistry for transition metal complexes. One major application there is the field of spin-crossover materials. The spin crossover effect is known, since over 80 years [13, 14], but with the discovery of the light-induced excited spin state trapping effect (LIESST-effect) [15, 16] in 1984, spin crossover complexes arouse nearly overnight notable interest as switchable components for data storage [17, 18], sensor materials [19], and miniaturization of magneto-optical devices [20, 21].

Two of the earliest publications about spin-transition in N1-tetrazole-complexes were the reports on 1-alkyltetrazole-complexes by Franke [22, 23]. Since these first publications, tetrazoles were used in many investigations, as variation of crystallization [24, 25], solvent [25], the anion and its size [26], spacer-substitution [27, 28] and length [28], or the number of coordinating sites [27, 28] provides independent different access–points allowing for rational design of the ligand system.

Different synthetic approaches towards N1-substituted tetrazoles were established so far. They are based on [2 + 3]-cycloaddition of isonitriles and azides [4–6], functionalization of 1*H*-tetrazole using tosylates [29], or a cycloaddition of sodium azide, triethyl orthoformate, and a primary amine known as the Franke-synthesis [23]. Although the Franke-synthesis is by far the most common, all approaches have, in common, moderate yields. A more challenging weakness of these approaches is the reagent introducing the substitution pattern: only a few isonitriles and tosylates are commercially available. Regarding the primary amines, the spectrum of commercial materials is notably larger, but many substrates need to be purified in advanced or—in the case of a non-available amine precursor—synthesized in the lab. Therefore, an approach using alternative substrates was attempted. Main focus points for this purpose were first, a larger commercial spectrum of available substitution patterns which are, in best case, also cheaper than amines. Second, the use of bench-stable and, if necessary, easily prepared precursors, and third, shorter reaction times and possibly higher yields in the tetrazole formation.

For this purpose, we decided to focus on the reaction of alkali tetrazolates (Tz) with alkyl halides.

## Results and discussion

The complete series of alkali-tetrazolate salts was reported in 2008 by Klapötke et al. [30]. All of these salts are relatively stable compounds and easily prepared in good yields by neutralization of a 1*H*-tetrazole solution with the corresponding base.

Regarding the alkylation reaction of tetrazoles, a non-negligible difficulty is found in its tendency towards isomerization: For many substitution reactions of tetrazoles, it is known that a mixture of the N1–N2 isomeric products is obtained [1].

To gain a first impression on the feasibility of the attempted alkylation reaction in a first screening series, 1-chlorobutane, 1-bromobutane, and 1-iodobutane were reacted for 6 h at 25, 75, and 150 °C with the lithium, sodium, and potassium salts of 1*H*-tetrazole. For the reaction, sealed vessels with THF as solvent were used (Scheme 2). The tetrazolate salts were prepared according to the literature. The isomer ratio is easily determined by ^1^H NMR, as the tetrazolic CH and the signals of the adjacent CH_x_-groups are significantly shifted to low field in the case of the N2-isomer.

**Figure:**
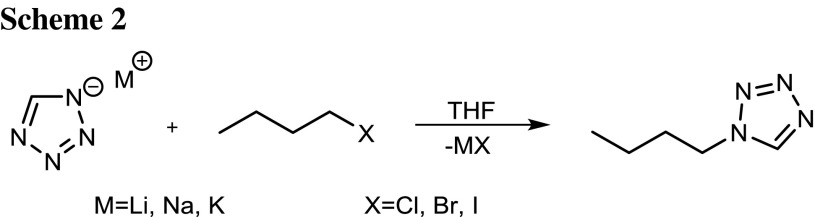

Whereas for room temperature no reaction was evidenced, for the samples at elevated temperatures, 1-butyl-1*H*-tetrazole as alkylation product was isolated. Independent from the used tetrazolate, for all reactions at 150 °C, 1:1 mixtures of the N1 and N2 isomer were identified. The best results were achieved at 75 °C, where for the first time, a clear tendency towards the desired N1 isomer was observed: LiTz yielded a 3:1 mixture of N1 and N2 isomer (NaTz and KTz both 1:1). It should be mentioned that in all experiments, the 1-iodobutane behaved worse than the corresponding chloride and bromide, as coloured products along untraceable by-products were isolated.

In a second series of experiments, LiTz was reacted with 1-bromobutane in methanol for 6 h at 130 °C in a sealed vessel in presence of 10 mol% LiOH as additional base and once as control experiment without. The presence of base turned out to be disadvantageous, as a 1:1.5 ratio of N1/N2 isomers was found. In contrast, the use of MeOH led to 49% isolated yield of 1-butyl-1*H*-tetrazole, contaminated by only 11% N2-isomer.

For a detailed screening of temperature, reaction time, and stoichiometry on basis of these first results, the combination of LiTz and 1–bromobutane in methanol was chosen. To decrease the reaction time and enlarge the temperature window, all further reactions were performed using a laboratory microwave oven.

As the amount of pure N1-alkylation was irreproducible under all investigated circumstances, the quality of the lithium tetrazolate was identified to be the only variable among these experiments. By determination of the pH-value and use of powder X-ray diffraction (P–XRD) analysis, for the so far used batches of LiTz, varying amounts of contamination with LiOH·H_2_O and Li_2_CO_3_ were found. As the used anhydrous LiOH is very sensitive towards moisture and atmospheric CO_2_, an alternative procedure for the LiTz-synthesis was developed. By the use of a freshly prepared LiOMe-solution in MeOH and subsequent removal of unreacted 1*H*-tetrazole by extraction with hot anhydrous isopropanol, a reproducibly pure LiTz could be obtained (Scheme 3).

**Figure:**
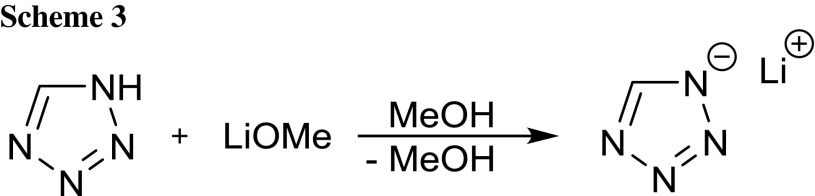

The use of high temperatures (varying conditions between 140 and 200 °C) and the use of methanol yielded rather irreproducible results, leading to a systematic optimization regarding reaction temperature and polarity of the solvent. To enhance reactivity and reduce reaction time, the addition of 10 mol% of lithium iodide hydrate was found to be advantageous. With constant yield and isomer ratio, the reaction temperature could be decreased to 100 °C and reaction times of 45 min. Samples reacted for longer times in the microwave showed increasing N2-isomer fractions.

**Fig. 1:**
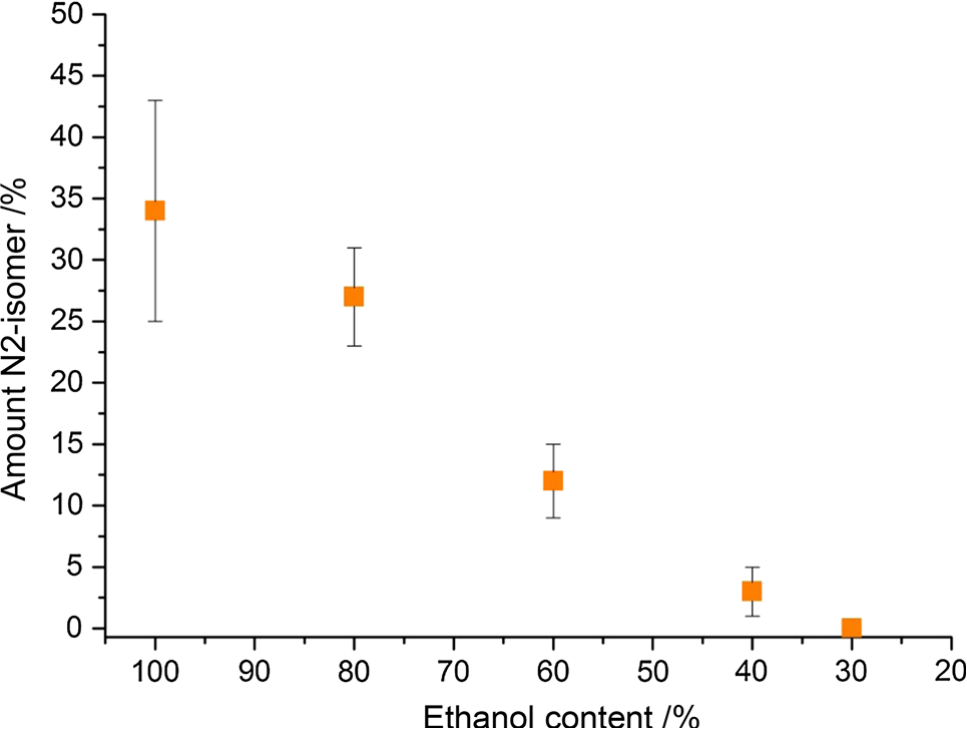
Correlation between N2-isomer concentration and ethanol–water ratio

Very promising results were obtained due to the variation of the solvent polarity. The use of anhydrous ethanol yielded reproducible mixtures with N2 contents between 20 and 43%. The use of water instead of methanol led to a negligible product formation, promoting the formation of intractable products. By the use of water–ethanol mixtures, finally, the desired selectivity could be obtained. As shown in Fig. 1, the amount of N2-isomer decreases with increasing water content. Using 30% v/v ethanol–water mixtures led, reproducibly, to pure N1-isomeric products. By means of NMR and IR spectroscopy, no N2-isomer formation was detected.

Summarizing the optimization procedure, the best results were achieved using a mixture of fresh lithium tetrazolate and R-Br substrate (1–3 mmol per cm^3^ of solvent) in a solution of 30 vol.% ethanol–water mixture, reacted for 45 min under microwave conditions at 100 °C.

Once proper conditions were found, the reaction was repeated with different substrates. Yields and structure of the investigated materials are given in Table 1.

**Table 1:**
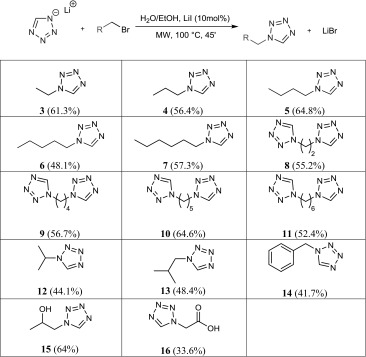
Target structures and isolated yields used in the substrate screening (conditions and used substrates are given in the "Experimental section")

For the selection of substrates presented in Table 1, the conditions developed during the screening-process at hand of the reaction of 1–bromobutane with LiTz were successfully applied. The formation of the corresponding N2-isomer was not evidenced in any case. Contrasting the newly developed substitution protocol with the so far commonly used Franke-synthesis, the yields could not be notably enhanced for all investigated cases. Moreover, the main advantages of the alkylation protocol are shorter reaction times and, especially, the use of bromides as cheap commercially available starting materials with a broader structural variety than for amines. The alkylation protocol cannot be seen as replacement for the Franke reaction, but notably enhances the flexibility regarding synthetic approaches to desired target structures.

## Conclusion

Based on the alkylation of alkali tetrazolates, within this work, a novel approach to N1-functionalized tetrazoles was developed. The aim was to establish a protocol using bench-stable commercial substrates, which are cheaper and available for a broader scope of substrates than amines are. These demands were satisfied by the alkylation of lithium tetrazolate with alkyl bromides.

The major difficulty for alkylation of the tetrazole was the N1–N2 isomerism. Within several screening steps, the purity of the lithium tetrazolate, as well as the polarity of the solvent were identified as crucial parameters. A modified protocol using lithium methanolate guarantees for a reproducibly high quality of the LiTz. In combination with 30 vol.% aqueous ethanol as solvent, this protocol allows for quantitative suppression of N2-isomer formation. The reaction was optimized using microwave conditions, leading finally to a protocol, which allows for good yields after 45-min reaction time at 100 °C. Whereas longer reaction times do not significantly increase the yield, they promote side reactions.

The alkylation reaction leads in a very short time to a broad spectrum of interesting ligands, as could be shown by several examples within this work. In summary, it was possible to modify, through this new approach, the ligand synthesis for N1-substituted tetrazoles in an effective way. This allows, in the future, for significantly higher throughput and an optimized design of interesting target structures for spin-crossover materials based on N1-substituted tetrazoles.

## Experimental

For all experiments, reagents and solvents were commercially obtained from Sigma-Aldrich and used as supplied. Unless otherwise stated, all reactions were performed under normal aerobic conditions.

^1^H and ^13^C{1H} NMR spectra were recorded on a Bruker Avance UltraShield 400 spectrometer with broad-band probe head. All NMR chemical shifts are reported in ppm; ^1^H and ^13^C shifts are referenced to the residual solvent resonance. Mid-range infrared spectra were recorded in ATR technique within the range of 4000–450 cm^−1^ using a PerkinElmer Spectrum Two FTIR spectrometer with an UATR accessory attached. If not otherwise stated, the background was measured with opened anvil versus ambient air. The powder X–ray diffraction measurements were carried out on a PANalytical X’Pert diffractometer in Bragg–Brentano geometry using Cu K_α1,2_ radiation, an X’Celerator linear detector with a Ni–filter, sample spinning with back loading zero background sample holders and 2*θ* = 4°–90°, *T* = 297 K at the X–ray Center at TU Wien. The diffractograms were evaluated using the PANalytical program suite HighScore Plus v3.0d. A background correction and a K_α2_ strip were performed.

*Warning: Tetrazoles and its derivatives are potential explosive and shock sensitive compounds; therefore handle with care. Proper protective measures should be taken *[31, 32].

### 1*H*-tetrazole (**1**)

NH_4_Cl (53.49 g, 1 mol, 1 eq.) and 97.51 g NaN_3_ (1.5 mol, 1.5 eq.) were suspended in 258 cm^3^ triethyl orthoformate (1.55 mol, 1.55 eq.) and 500 cm^3^ acetic acid. The reaction mixture was stirred for 18 h at 95 °C and filtrated after cooling. After evaporation to dryness, the solid residue was extracted with 450 cm^3^ of boiling isopropanol. This step was repeated once. The off-white residue after evaporation of the combined isopropanol fractions was recrystallized from ethanol. Yield: 42.4 g (60.5%). The spectroscopic properties (^1^H NMR, ^13^C{1H} NMR, and MIR) were found to be identical with the ones described in Refs. [30].

### Lithium tetrazolate (**2**)

Lithium granulate (2 g, 0.29 mol, 1 eq.) was dissolved under cooling in 250 cm^3^ dry methanol. To the lithium methoxide solution, slowly, 22.2 g of 1*H*-tetrazole (0.32 mol, 1.1 eq.) was added. The suspension was stirred for 1 h at room temperature. After filtration, the lithium tetrazolate was dried in vacuum and stored under inert gas. Yield: 18.3 g (83.6%). The spectroscopic properties (^1^H NMR, ^13^C{1H} NMR, and MIR) were found to be identical with the ones described in Ref. [30].

### General procedure for microwave alkylations

Freshly prepared lithium tetrazolate (228 mg, 3 mmol, 1 eq.) and 3 mmol of the alkyl bromide were dissolved in 3 cm^3^ of 30% v/v ethanol in a microwave vial. Lithium iodide hydrate (45.5 mg, 0.3 mmol, 0.1 eq.) was added. The mixture was immediately reacted in the microwave at 100 °C for 45 min. Two different workup strategies were used:

*Workup A* The reaction mixture was diluted with 5 cm^3^ water and, afterwards, extracted twice with 10 cm^3^ of hexane to remove the eventually formed N2-isomer. Afterwards, the extraction was repeated three times with 10 cm^3^ of ethyl acetate each. The combined organic phases were dried over MgSO_4_ and evaporated to yield the product.

*Workup B* The reaction mixture was filtered through a thin layer of silica gel and evaporated. The solid residue was triturated with 30 cm^3^ ethyl acetate and evaporated, yielding the product.

### Indicated yields refer to isolated yields

#### 1-Ethyl-1*H*-tetrazole (**3**)

Lithium tetrazolate and 1-bromoethane were used as reagents. Workup A, product obtained as colourless oil. Yield: 180.43 mg (61.3%). The spectroscopic properties (^1^H NMR, ^13^C{1H} NMR, and MIR) were found to be identical with the ones described in Ref. [28].

#### 1-Propyl-1*H*-tetrazole (**4**)

Lithium tetrazolate and 1-bromopropane were used as reagents. Workup A, product obtained as colourless oil. Yield: 189.74 mg (56.4%). The spectroscopic properties (^1^H NMR, ^13^C{1H} NMR, and MIR) were found to be identical with the ones described in Ref. [28].

#### 1-Butyl-1*H*-tetrazole (**5**)

Lithium tetrazolate and 1-bromobutane were used as reagents. Workup A, product obtained as colourless oil. Yield: 245.26 mg (64. %). The spectroscopic properties (^1^H NMR, ^13^C{1H} NMR, and MIR) were found to be identical with the ones described in Ref. [28].

#### 1-Pentyl-1*H*-tetrazole (**6**)

Lithium tetrazolate and 1-bromopentane were used as reagents. Workup A, product obtained as colourless oil. Yield: 202.29 mg (48.1%). The spectroscopic properties (^1^H NMR, ^13^C{1H} NMR, and MIR) were found to be identical with the ones described in Ref. [33].

#### 1-Hexyl-1*H*-tetrazole (**7**)

Lithium tetrazolate and 1-bromohexane were used as reagents. Workup A, product obtained as colourless oil. Yield: 265.11 mg (57.3%). The spectroscopic properties (^1^H NMR, ^13^C{1H} NMR, and MIR) were found to be identical with the ones described in Ref. [33].

#### 1,2-Bis(tetrazol-1-yl)ethane (**8**)

Lithium tetrazolate and 1,2-dibromoethane were used as reagents. Workup B, product obtained as white solid. Yield: 275.14 mg (55.2%). The spectroscopic properties (^1^H NMR, ^13^C{1H} NMR, and MIR) were found to be identical with the ones described in Ref. [34].

#### 1,4-Bis(tetrazol-1-yl)butane (**9**)

Lithium tetrazolate and 1,4-dibromobutane were used as reagents. Workup B, product obtained as off-white solid. Yield: 330.33 mg (56.7%). The spectroscopic properties (^1^H NMR, ^13^C{1H} NMR, and MIR) were found to be identical with the ones described in Ref. [35].

#### 1,5-Bis(tetrazol-1-yl)pentane (**10**)

Lithium tetrazolate and 1,5-dibromopentane were used as reagents. Workup B, product obtained as white solid. Yield: 403.55 mg (64.6%). The spectroscopic properties (^1^H NMR, ^13^C{1H} NMR, and MIR) were found to be identical with the ones described in Ref. [36].

#### 1,6-Bis(tetrazol-1-yl)hexane (**11**)

Lithium tetrazolate and 1,6-dibromohexane were used as reagents. Workup B, product obtained as beige solid. Yield: 349.39 mg (52.4%). The spectroscopic properties (^1^H NMR, ^13^C{1H} NMR, and MIR) were found to be identical with the ones described in Ref. [24].

#### 1-Isopropyl-1*H*-tetrazole (**12**)

Lithium tetrazolate and 2-bromopropane were used as reagents. Workup A, product obtained as colourless oil. Yield: 148.36 mg (44.1%). The spectroscopic properties (^1^H NMR, ^13^C{1H} NMR, and MIR) were found to be identical with the ones described in Ref. [37].

#### 1-Isobutyl-1*H*-tetrazole (**13**)

Lithium tetrazolate and 1-bromo-2-methylpropane were used as reagents. Workup A, product obtained as colourless oil. Yield: 183.18 mg (48.4%). The spectroscopic properties (^1^H NMR, ^13^C{1H} NMR, and MIR) were found to be identical with the ones described in Ref. [37].

#### 1-Benzyl-1*H*-tetrazole (**14**)

Lithium tetrazolate and (bromomethyl)benzene were used as reagents. Workup B, product obtained as white solid. Yield: 200.39 mg (41.7%). The spectroscopic properties (^1^H NMR, ^13^C{1H} NMR, and MIR) were found to be identical with the ones described in Ref. [38].

#### 1-(1*H*-Tetrazol-1-yl)propan-2-ol (**15**)

Lithium tetrazolate and 1-bromopropan-2-ol were used as reagents. Workup A, product obtained as colourless oil. Yield: 219.09 mg (64%). The spectroscopic properties (^1^H NMR, ^13^C{1H} NMR, and MIR) were found to be identical with the ones described in Ref. [1, 4].

#### 2-(1*H*-Tetrazol-1-yl)acetic acid (**16**)

Lithium tetrazolate and 2-bromoacetic acid were used as reagents. Workup B, product obtained as white solid. Before evaporation of the crude reaction mixture, the solution was acidified using hydrochloric acid. For the final extraction, isopropanol instead of ethyl acetate was used. Yield: 129.11 mg (33.6%). The spectroscopic properties (^1^H NMR, ^13^C{1H} NMR, and MIR) were found to be identical with the ones described in Ref. [39].
